# Study of physico-chemical properties and in vitro antimicrobial activity of hydroxyapatites obtained from bone calcination

**DOI:** 10.1007/s40204-018-0105-2

**Published:** 2018-12-31

**Authors:** Cássio M. Resmim, Mariane Dalpasquale, Nilce I. C. Vielmo, Filipe Q. Mariani, Juan C. Villalba, Fauze J. Anaissi, Mirian M. Caetano, Marcelo M. Tusi

**Affiliations:** 1 Universidade Regional Integrada do Alto Uruguai e das Missões–URI, Av. Batista Bonoto Sobrinho, 733, São Vicente, Santiago, RS 97700-000 Brazil; 2 Universidade Estadual do Centro-Oeste–UNICENTRO, R. Simeão Varela de Sá, 03, Vila Carli, Guarapuava, PR 85040-080 Brazil; 3 Faculdade Campo Real, R. Comendador Norberto, 1299, Santa Cruz, Guarapuava, PR 85040-080 Brazil

**Keywords:** Hydroxyapatite, *Staphylococcus aureus*, Biomaterial, Bovine, Porcine

## Abstract

Hydroxyapatite was obtained by bone calcinations. To study the calcination process, bovine and porcine bones were first autoclaved to remove fat and other non-bone tissues. They were then heated in an alumina pan in an oxidizing atmosphere of air, where simultaneous thermal analysis curves were recorded. To prepare the hydroxyapatites, bone samples were calcined at 850 °C and 1000 °C using a muffle furnace for 1 h. The obtained materials were powdered using mortar and pestle, and sifted in a sieve (60 mesh) without any additional purification or chemical treatment. The materials obtained were characterized by energy-dispersive X-ray spectroscopy, X-ray diffraction, and Fourier-transform infrared spectroscopy. The antimicrobial properties of these materials were determined through direct contact tests against *Staphylococcus aureus*. The natural hydroxyapatites obtained by bone calcination inhibited *S. aureus* growth, with the material obtained by calcination of bovine bones at 1000 °C, showing the best antimicrobial activity. These results indicated that bone wastes can be used to obtain hydroxyapatites with antimicrobial activity.

## Introduction

Bones of vertebrate animals are composed of hydroxyapatites (30–70 wt%), collagen, Na^+^, Mg^+2^, and CO_3_^−2^ ions, and other minor compounds. The stoichiometric hydroxyapatite can be chemically synthesized, and it has formula Ca_10_(PO_4_)_6_(OH)_2_ and Ca/P atomic ratio of 1.67, although other stable hydroxyapatites can also have different Ca/P ratios (Francis and Webb 1970; Dorozhkin and Epple 2002; Pebla et al. 2014; Abou Neel et al. 2016). This compound belongs to the calcium orthophosphate family, and it is the less soluble compound of its class in physiological aqueous environments. It has bioactive, biocompatible, and osteoconductive properties (Dorozhkin and Epple 2002; Barakat et al. 2008; Tan et al. 2013). The application of hydroxyapatite and calcium phosphate as biomaterials has been widely studied (Itoh et al. 2005; Oliveira et al. 2009; Tan et al. 2013; Zakaria et al. 2013; Shi et al. 2017).

Bacterial growth reduces the functionality and effectiveness of biomaterials as implants. In this context, the prevention of bacterial infections after the placement of an implant is crucial. Biomaterial-associated infections are generally resistant to antibiotics, and the removal of an infected implant is often the only possible solution, which, however, generates high costs for the health-care system and discomfort for the patient (Li and Webster 2018). Antibiotics are thus critical for the success of orthopedic prosthetic surgeries. Antibiotic resistance is occurring in nearly all the bacteria, including the bacteria that most commonly cause orthopedic infections, such as *S. aureus* (Inzana et al. 2016; Li and Webster 2018). In arthroplasties, the infection rates are unfortunately high, and cause devastating effects on patients, such as reduction of their lifespan and increase in the failure rates of prostheses. Infections are also responsible for enormous medical costs, increase in morbidity, and patient dissatisfactions (Chen et al. 2007). Thus, it is important to determine the antimicrobial properties of biomaterials, especially against *Escherichia coli* e *Staphylococcus aureus*, as these are the most common source of contamination of prosthetic implants (Feitosa et al. 2016).

The physico-chemical and, consequently, antimicrobial properties of hydroxyapatite can be affected by synthesis conditions, namely reagent addition rate, reaction temperature, and others (Koutsopoulos 2002; Janusz and Skwarek 2009; Kehoe et al. 2011). Hydroxyapatite is commonly prepared by wet precipitation (Monmaturapoj 2008; Abidi and Murtaza 2014; Feitosa et al. 2016), sol–gel (Sanosh et al. 2009; Costescu et al. 2010; Agrawal et al. 2011), and hydrothermal method (Earl et al. 2006; Feitosa et al. 2016). However, the use of calcination (Mkukuma et al. 2004; Figueiredo et al. 2010; Fara and Abdullah 2016) and chemical treatment to obtain hydroxyapatite (Barakat et al. 2008) has also been reported. The extraction of hydroxyapatite from the bio-wastes is both a biologically safe (no external chemical is required) and economically desirable process (Barakat et al. 2008). Many reports describe the use of bio-wastes such as fish bones (Ozawa and Suzuki 2002; Fara and Abdullah 2016), bovine bones (Joschek et al. 2000), and bones and teeth of pigs (Xiaoying et al. 2007) to obtain hydroxyapatite.

In this work, nanostructured hydroxyapatites were obtained by calcination of bovine and porcine bones at two different temperatures. The obtained materials were characterized by X-ray diffraction (XRD), scanning electronic microscope (SEM), energy-dispersive spectroscopy (EDX), Fourier-transform infrared (FTIR), and the antimicrobial properties were evaluated against *S. aureus*. While the extraction of hydroxyapatite from bio-wastes has been largely explored, the antimicrobial properties of these materials and/or the influence of calcination temperature on the antimicrobial properties have so far remained elusive.

## Experimental

### Bone collection and preparation

Bovine and porcine bones were collected in supermarket butcher departments and kept cold in a refrigerator without any further treatment. The bones came from the diaphysis of bovine and porcine femur. To prepare hydroxyapatite, bones were first autoclaved for 1 h at one atmosphere to eliminate aggregated tissues and organic compounds (mostly fat and blood). Subsequently, fat and other non-bone tissues were manually removed and the bones were dried at 50 °C (Miyahara et al. 2007).

### Study of bone calcination

To study the calcination of bones, simultaneous thermal analysis was performed using a Seiko SII Exstar 6000. Samples of bovine and porcine bones (about 15 mg) were heated in an alumina pan, with temperature increasing at a rate of 10 °C/min from 25 to 1000 °C in an oxidizing atmosphere of air (Mondal et al. 2012).

#### Hydroxyapatite preparation by bone calcination

To obtain the hydroxyapatites, the bone samples (about 25 g) were calcined at 850 and 1000 °C (at a rate of 10 °C/min) for 1 h using a Jung (model 0812) muffle furnace and static atmosphere (Miyahara et al. 2007; Figueiredo et al. 2010; Fara and Abdullah 2016). The materials were powdered using a mortar and pestle, and sifted in a 60-mesh sieve.

### Characterization and chemical analysis

The Ca/P ratio analysis of bone-derived hydroxyapatites was carried out using an energy-dispersive X-ray fluorescence spectrometry (EDXRF) instrument (Shimadzu, model EDX 8000). A spectral resolution of 132.58 eV for Mn K_α_ was achieved, and the maximum count was 1088.5417 cps/μA.

The X-ray diffraction analyses were performed using a Bruker diffractometer model D2 Phaser with CuKα radiation source (*λ* = 0.15406 nm). The XRD was recorded from 2*θ* = 10–70° with a step size of 0.05° and a scanning time of 2 s per step. The crystallinity (*X*_C_), corresponding to the fraction of crystalline phase present in the examined volume, was evaluated by the relation:

1 $$\chi_{\text{C}} \approx 1 - \left( {\frac{{V_{112/300} }}{{I_{300} }}} \right),$$

where *I*_300_ is the intensity of (300) reflection and *V*_112/300_ is the intensity of the hollow between (112) and (300) reflections, which completely disappears in non-crystalline samples (Landi et al. 2000; Figueiredo et al. 2010). The mean crystallite size (*d*) was calculated using the Scherrer equation:

2 $$d = \frac{K\lambda }{{B_{2\theta } \cos \theta }}.$$

In this expression, *λ* is the wavelength of the radiation, *θ* is the Bragg angle, and B_2*θ*_ indicates the line broadening at half the maximum intensity of the (002) reflection (Figueiredo et al. 2010).

The Fourier-transform infrared spectroscopy spectra were recorded on a Thermo Nicolet IR200 spectrometer in the wavenumber range 800–3700 cm^−1^. To prepare pellets, samples of the hydroxyapatite were first powdered in an agate mortar and then mixed with potassium bromide (KBr). The mixture was pressed, while the system was evacuated with an oil pump.

### Antibacterial activity

The antibacterial activity of the obtained hydroxyapatites against *S. aureus* (strain NEWP0023) was measured by direct contact in a solid medium, as previously described (Feitosa et al. 2016). Briefly, a bacterial suspension (10^5^ cfu/mL) was prepared in saline solution (NaCl 0.85%). Then, 2000 μL of this suspension was transferred to an Eppendorf tube containing 10 mg of the material, stirred, and then, 100 μL of the mixture was spread onto agar plates. The plates were incubated at 37 °C for 24 h. After this period, the number of bacterial colonies on each plate was counted. As growth-positive control, the bacterial suspension spreads onto agar plates in the absence of materials. All trials were performed in triplicate, and the results were normalized by the calculation of the arithmetic average. The inhibitory effect produced by each test material was calculated according to the equation:

3 $${\text{IF}} = \left( {\frac{{C_{\text{control}} - C_{\text{test}} }}{{C_{\text{control}} }}} \right) \times 100\% ,$$

where IF stands for the inhibitory effect, *C*_control_ is the arithmetic average of the colony-forming units grown on control plates, and *C*_test_ is the arithmetic average of the colony-forming units grown on test plates.

## Results and discussion

### Study of bone calcination

**Fig. 1:**
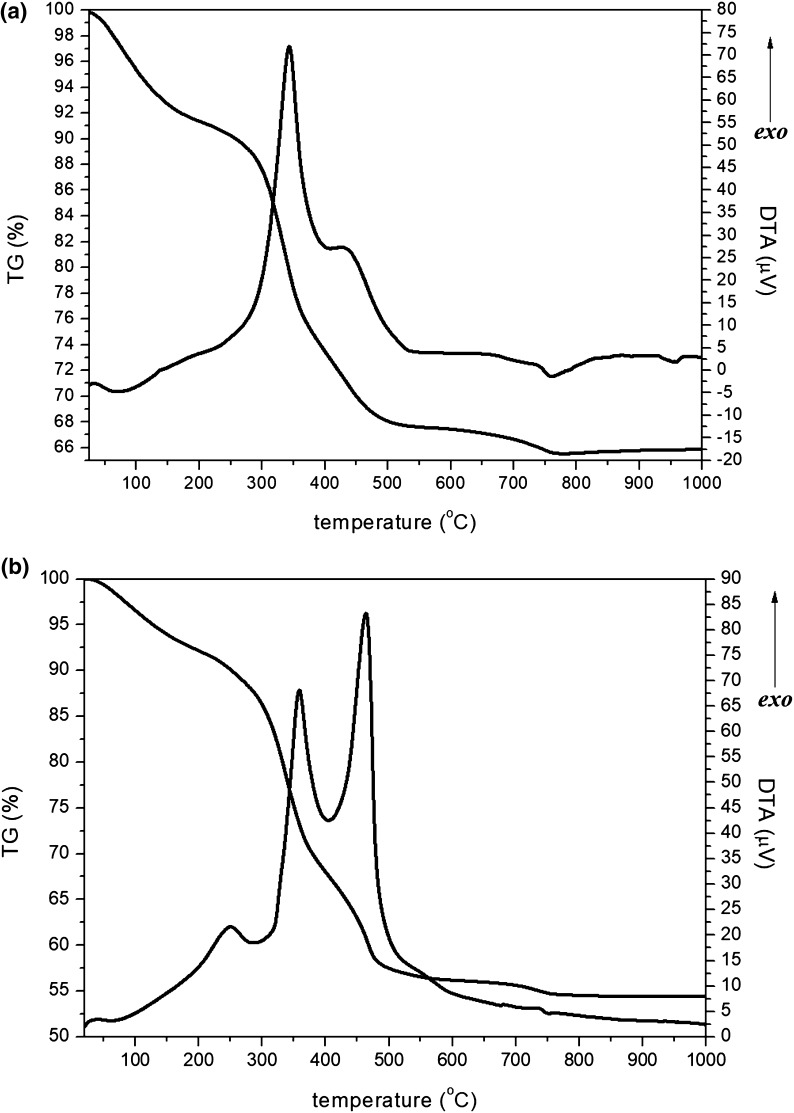
Thermal curves obtained by simultaneous thermal analysis for the process of calcination of bones: **a** bovine and **b** porcine

The thermal curves of the bone calcination process were obtained by simultaneous thermal analysis, as shown in Fig. 1. The thermal curves of the samples typically showed the occurrence of three successive processes of weight loss: the first below 200 °C, the second between 200 and 600 °C, and the third between 600 and 800 °C. Above 800 °C, the weight loss was negligible. The first process was attributed to the dehydration of the bone, also referred to as loss of surface water (Haberko et al. 2006; Figueiredo et al. 2010; Miculescu et al. 2012). The second process observed in the thermal curves was associated with the combustion of the bone organic matter, mainly collagen (Figueiredo et al. 2010; Miculescu et al. 2012). The third process of weight loss observed in the thermal curves was associated with the process of carbon dioxide release (CO_2_), due to the decomposition of the carbonate ions (CO_3_^−2^) present in the lattice of hydroxyapatite (Haberko et al. 2006; Murugan et al. 2006; Figueiredo et al. 2010). The natural hydroxyapatite is a non-stoichiometric compound and contains in its lattice carbonate ions which substitute for hydroxyl ions. Phosphate ions lie on the surface of the crystal. Carbonate ions are the most abundant substitute in bone minerals (approximately 3–8 wt%) (Murugan et al. 2006; Figueiredo et al. 2010). The data obtained in this work agree with the previous reports (Figueiredo et al. 2010), which showed the decomposition of carbonate ions at temperatures near 700 °C.

On the other hand, according to the reports by Murugan et al. (2006), carbon dioxide is released during the bovine bone calcination in an air atmosphere which occurs at temperatures between 400 and 600 °C. The loss of most carbonate groups present in the carbonated hydroxyapatite, which naturally occurs during its extraction from bio-wastes by heating, is a problem which affects the biological properties of the final product (Barakat et al. 2008). The main results obtained by thermogravimetric analysis are presented in Table 1.

**Table 1:** Amount of the main components of samples of bovine and porcine bones estimated using the thermal curves and Ca/P ratio of hydroxyapatite obtained after the thermogravimetric analysis

Attribution	Bovine	Porcine
H_2_O (wt%) (*T* < 200 °C)	8.7	8.2
Collagen (wt%) (200 < *T* < 600 °C)	26.2	39.1
Hydroxyapatite (wt%)^a^	65.1	52.7
CO_3_^−2^ (wt%)^b^ (600 °C < *T* < 800 °C)	2.8	3.0

^a^% = 100 − (%H_2_O + %collagen)

^b^% = [(*w*_600 °C_ − *w*_800 °C_)/*w*_600 °C_] × 100%

^c^Ca/P ratio of clear bone before calcination

The data in Table 1 indicate that the bovine and porcine bones have similar water content, but the proportion of organic matter is slightly different. Strikingly, the profile of the curve obtained in differential thermal analysis indicates that fat can still be present in the porcine bones used in the experiment. This fat increases the amount of organic matter and, therefore, the collagen content indicated in Table 1 is to be considered as both collagen and fat, resulting in a lower yield of calcination. The carbonate ion content was about 3% in both bones.

The differential thermal analysis curves for bovine bones (Fig. 1) showed an exothermic peak with a maximum at 77 °C and an accentuated exothermic peak with a maximum at about 330 °C and a shoulder at 430 °C. For the porcine bone, it was possible to observe an exothermic peak at about 117 °C, an endothermic peak at 89 °C, an exothermic peak at 246 °C, and two accentuated exothermic peaks at 339 and 463 °C. Thus, compared to the bovine bone, the porcine bone presented a peak rather than a shoulder, possibly due to higher organic content. Both samples presented an exothermic peak within 720–750 °C range.

### Hydroxyapatite preparation

Table 2 shows the properties of materials obtained by the calcination of bovine and porcine bones in a muffle furnace. The hydroxyapatite prepared by calcination of bones presented yield values in the range 59–66 wt%. These values were independent of the calcination temperature, but the yields depended on the source of bone. The calcination of porcine bones resulted in lower yield, possibly due to the higher fat content and porosity of this bone.

**Table 2:** The yield of calcination, Ca/P ratio, mean crystallite size, and crystallinity of materials obtained by the calcination of bones in a muffle furnace

Properties	Bovine		Porcine	
	850 °C	1000 °C	850 °C	1000 °C
Yield (wt%)	65	65	59	59
Ca/P ratio	2.02	1.98	2.00	1.96
Crystallite (nm)	19	19	22	19
Crystallinity	0.70	0.84	0.24	0.70
Antimicrobial activity (%)	30	81	38	56

### EDX analysis

The materials were obtained by bone calcination presented Ca/P ratio values between 1.96 and 2.02, higher than stoichiometric hydroxyapatite (1.67). Other authors (Joschek et al. 2000; Haberko et al. 2006) reported similar results. Higher Ca/P ratio values compared to the stoichiometric hydroxyapatite may be due to the occurrence of either calcium and phosphorus compounds other than hydroxyapatite or Ca compounds deprived of phosphorus (e.g., formation of CaO or CaCO_3_) (Francis and Webb 1970; Barakat et al. 2008;Figueiredo et al. 2010; Doostmohammadi et al. 2011; Bano et al. 2017). Besides the presence of calcium (Ca) and phosphorus (P), EDX analysis showed the presence of aluminum (Al), strontium (Sr), silicon (Si), potassium (K), zinc (Zn), and cooper (Cu), in agreement with previously published studies (Miculescu et al. 2012; Pebla et al. 2014).

### X-ray diffractometry

**Fig. 2:**
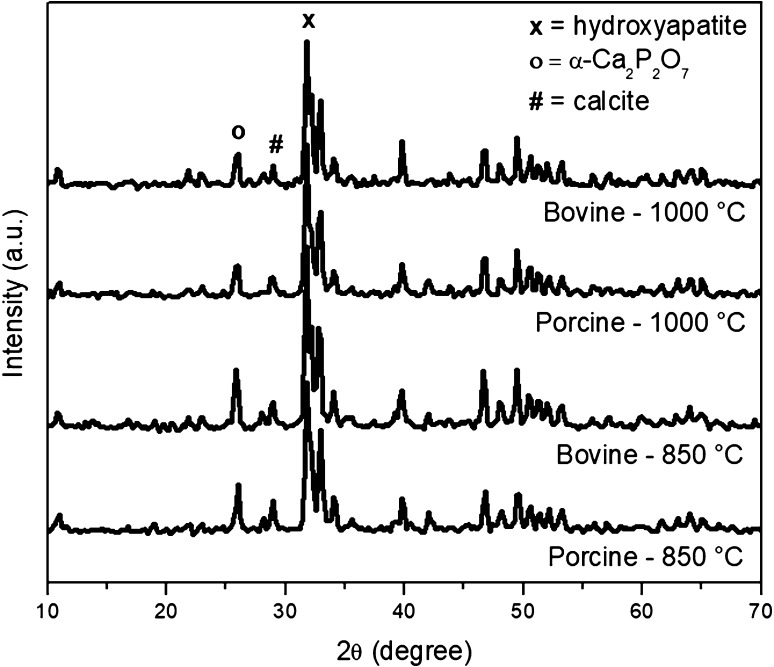
X-ray diffractograms of nanohydroxyapatite samples obtained by calcination of bones

Figure 2 presents the X-ray diffractograms of bone calcination products. All the obtained materials presented hydroxyapatite as the main phase. All the obtained materials presented peaks associated with calcite phase (ICDD 5-586). The hydroxyapatites obtained from bovine bones (ICDD 84-1998) had a different lattice structure than the hydroxyapatites obtained from porcine bones (ICDD 73-1731). Apparently, the materials obtained from bovine bones showed peaks associated with the α-Ca_2_P_2_O_7_ phase (ICDD 9-345), while materials obtained from porcine bones presented hydroxyapatite as the only phase. The formation of secondary phases, as observed, is associated with the degradation of hydroxyapatite due to high temperature. An example is a process in the range of temperature between 600 and 800 °C, associated with the decomposition of the carbonate ions present in the hydroxyapatite lattice (Landi et al. 2000; Miculescu et al. 2012).

The values of crystallinity degrees (Table 2) were in the range 0.24–0.84%. The lower crystallinity was registered for the material obtained by the calcination of porcine bones at 850 °C, while the higher crystallinity was observed for the material obtained by calcination of bovine bones at 1000 °C. This result is in accordance with other studies, which report that, in the extraction of hydroxyapatite by calcination of bones, mean particle size and crystallinity increase with the increase in the temperature of calcination (Figueiredo et al. 2010; Fara and Abdullah 2016). The materials obtained by calcination of bovine bones presented mean crystallite size of 19 nm, while the materials obtained by calcination of porcine bones showed mean crystallite size of 22 nm for the calcination temperature of 850 °C and 19 nm for the calcination temperature of 1000 °C. Mean crystallite size was calculated using the Scherrer formula.

### Infrared spectra

**Fig. 3:**
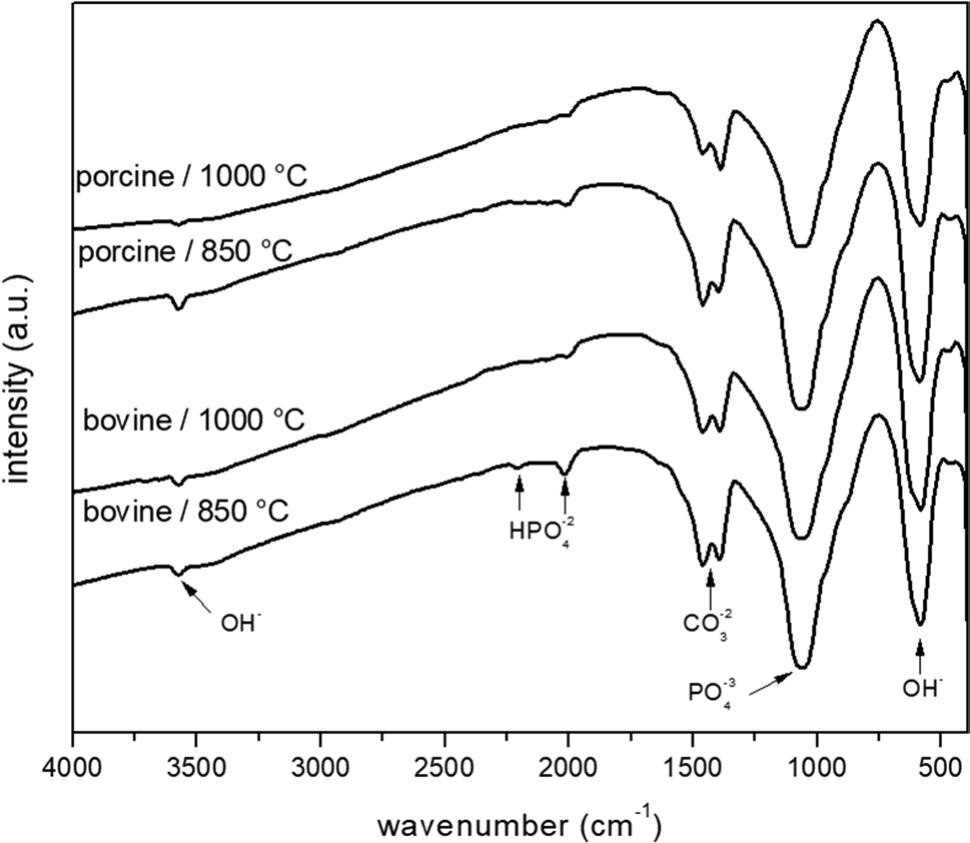
FTIR spectra of nanohydroxyapatite samples obtained by calcination of bones

To characterize the chemical groups in hydroxyapatite obtained by calcination of bones, an FTIR analysis was performed (Fig. 3). All FTIR spectra presented the same peaks, independent of bone used and temperature of calcination. The observed bands corresponded to hydroxyl (~ 3571 cm^−1^), phosphate (1057 cm^−1^ and 578 cm^−1^), and carbonate (1396 and 1458 cm^−1^) (Rau et al. 2004; Figueiredo et al. 2010; Laranjeira et al. 2016; Petrova et al. 2016). The presence of carbonate bands in the FTIR spectroscopy may be explained by residual carbonate ions that were not removed during the calcination process, corroborating the X-ray diffraction data. However, the incomplete elimination of carbonate ions contradicts the results of thermal analysis and is probably due the technical reason. In detail, the experiment in muffle furnace requires a larger bone piece (and, thus, higher bone weight) compared to the thermal analysis and, consequently, it is likely that the time used in thermal treatment to prepare the hydroxyapatite was not enough to eliminate the carbonate in the muffle furnace. The interpretation of the remaining bands (2013 and 2206 cm^−1^) is at the moment still under investigation. An interesting idea, according to Rau and coworkers, is that these bands are by-products of processes of association, liberation, or interaction with molecules polluting the matrix (Rau et al. 2004).

### Antibacterial activities

**Fig. 4:**
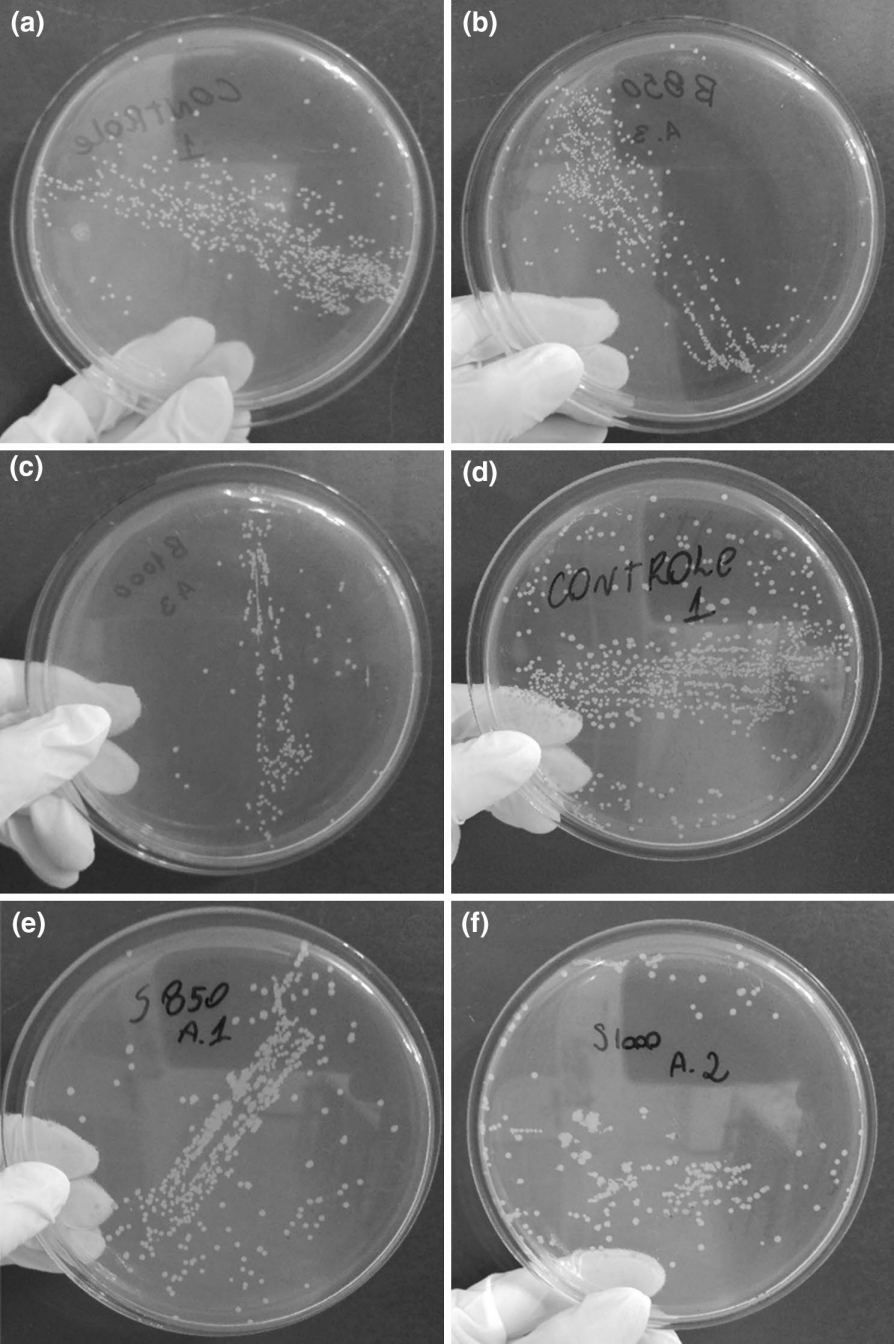
Determination of the antimicrobial activity of natural hydroxyapatites against *S. aureus*. **a** control for determination of antimicrobial activity of hydroxyapatites obtained from bovine bone; **b** hydroxyapatite obtained from calcination of bovine bone at 850 °C; **c** hydroxyapatite obtained from calcination of bovine bone at 1000 °C; **d** control for determination of antimicrobial activity of hydroxyapatites obtained from porcine bone; **e** hydroxyapatite obtained from calcination of porcine bone at 850 °C; **f** hydroxyapatite obtained from calcination of porcine bone at 1000 °C

Figure 4 presents images of cultures of *S. aureus,* used to determine the antimicrobial activity of the hydroxyapatites obtained in this study. Bacteria incubated in the presence of hydroxyapatite formed less colonies than the control (bacteria incubated in the absence of the tested material), suggesting a possible inhibitory effect of the hydroxyapatite on the growth of *S. aureus*. The materials obtained from bovine bones at 850 and 1000 °C showed a growth inhibitory effect of 30% and 81%, respectively, while the hydroxyapatites obtained by calcination of porcine bones at 850 and 1000 °C were able to inhibit 38% and 56% of the bacterial cells, respectively.

These data are in agreement with the results of other authors (Feitosa et al. 2016) that observed inhibitions of 97% and 95% for the *S. aureus* growth using powdered hydroxyapatites prepared by co-precipitation and the hydrothermal method, respectively. On the other hand, others (Predoi et al. 2016) reported that a thin film of pure hydroxyapatite deposited on Si disks did not show any antimicrobial activity against *S. aureus*. It is likely that the presentation of the tested material influences the results in antimicrobial activity tests, possibly due to the solubility of the hydroxyapatite. A variety of methods of determining antimicrobial activity have been studied and the results obtained are profoundly influenced by the method selected, microorganisms used to carry out the test, and the degree of solubility of each test compound (Valgas et al. 2007). It is known that hydroxyapatite is an insoluble material, especially in an alkaline medium (Semdé et al. 2012). The Müeller–Hinton medium and phosphate-buffer saline (PBS) have a pH close to 7.2. Therefore, the hydroxyapatite is practically insoluble in these media and its diffusion rate is very small. In the case of a thin film of pure hydroxyapatite deposited on a Si disk, it is likely that the interaction between the ceramic material and the microorganisms is insufficient to inhibit bacterial growth. Instead, in the case of powdered hydroxyapatite, the surface area is larger, increasing the interaction between the material and the bacteria and, consequently, increasing the inhibitory effect.

Gram-positive bacteria such as *S. aureus* have a cell wall composed of a thick layer of peptidoglycan, a polymer of carbohydrates and charged amino acids, which makes them highly hydrophilic (Harris et al. 2002; Ragab et al. 2014; Feitosa et al. 2016). The antibacterial activity observed for hydroxyapatite against *S. aureus* may be associated with the hydrophilic nature of this material due to the presence of hydroxyl groups, which may favor the interaction with the cell wall of Gram-positive bacteria (Feitosa et al. 2016). The antimicrobial activity of hydroxyapatite can be attributed to the production of reactive oxygen species (OH^−^, H_2_O_2_, and O_2_^−2^) on the surface of the hydroxyapatite nanoparticles, linked with fatal damage to the bacteria. Another possible explanation for the antibacterial effect is that the abrasive surface ordering (i.e., texturing) of the hydroxyapatite due to surface defects and aggregates can contribute to the mechanical damage to the bacterial cell membrane (Ragab et al. 2014).

## Conclusions

Nanostructured hydroxyapatite was prepared by calcination of bovine and porcine bones at different temperatures with good yield. The hydroxyapatites obtained by calcination of bones showed a mixture of phases containing hydroxyapatite and calcium carbonate phases. FTIR spectra indicated that all materials presented peaks associated with phosphate, hydroxyl, and carbonate groups. The Ca/P ratio values of obtained materials were acceptable for hydroxyapatite materials, but these values were higher compared to the theoretical values, probably due to the formation of the calcite phase. The natural hydroxyapatites obtained by calcination of bovine and porcine bones could inhibit *S. aureus* growth, with the material prepared by calcination of bovine bones at 1000 °C presenting an antibacterial activity against *S. aureus* superior compared to other materials. The antimicrobial activity was weakly influenced by the source of bone used, and higher calcination temperatures led to greater inhibitions of *S. aureus* growth.
